# GEO-LIFE COURSE DETERMINANTS OF EDUCATIONAL DISPARITIES IN U.S. ADULT HEALTH

**DOI:** 10.1093/geroni/igz038.220

**Published:** 2019-11-08

**Authors:** Blakelee Kemp, Blakelee R Kemp, Jennifer K Montez

**Affiliations:** Syracuse University, Syracuse, New York, United States

## Abstract

Educational attainment is one of the strongest social determinants of adult health. However, recent studies show that it is a stronger determinant in some areas of the country than others. This study investigates geographic and life course contexts that may explain the pattern. We merge data on adults aged 50+ in the Health and Retirement Study (1998-2014) with contextual data on their state(s) of birth and residence. We examine: (1) how the education-health association varies across regions, and (2) how childhood (e.g., poverty, compulsory schooling) and adulthood experiences (e.g., smoking, minimum wage) explain the variation. Findings reveal that the education-health association varies across regions and is more pronounced for outcomes further along in the disablement process. Poor childhood health, adult behaviors, and states’ economic policies partly explain why the association varies across regions. The findings underscore the importance of geographic and life course contexts for understanding educational disparities in health.

